# Benzoximate

**DOI:** 10.1107/S1600536812044248

**Published:** 2012-10-31

**Authors:** Tae Ho Kim, Suk-Hee Moon, Jineun Kim, Ki-Min Park

**Affiliations:** a Department of Chemistry and Research Institute of Natural Sciences, Gyeongsang National University, Jinju 660-701, Republic of Korea; b Department of Food and Nutrition, Kyungnam College of Information and Technology, Busan 617-701, Republic of Korea

## Abstract

In the title compound [systematic name: (3-chloro-2,6-dimeth­oxy­phen­yl)(eth­oxy­imino)­methyl benzoate], C_18_H_18_ClNO_5_, the phenyl and chloro­dimeth­oxy­phenyl rings are linked by the eth­oxy­imino­methyl benzoate system such that they are almost perpendicular to each other with the dihedral angle between them being 85.72 (9)°. In the crystal, C—H⋯O and C—H⋯Cl hydrogen bonds between the phenyl and chloro­dimeth­oxy­phenyl rings generate *R*_2_^2^(8) rings which link the mol­ecules into zigzag chains along the *b* axis. Additional C—H⋯O contacts, together with weak inter­molecular C—H⋯π inter­actions, further link the mol­ecules into a three-dimensional network.

## Related literature

For information on the toxicity of the title compound, see: Kim *et al.* (2007 ▶). For a description of the Cambridge Structural Database, see: Allen (2002 ▶). For hydrogen-bond motifs, see: Bernstein *et al.* (1995 ▶).
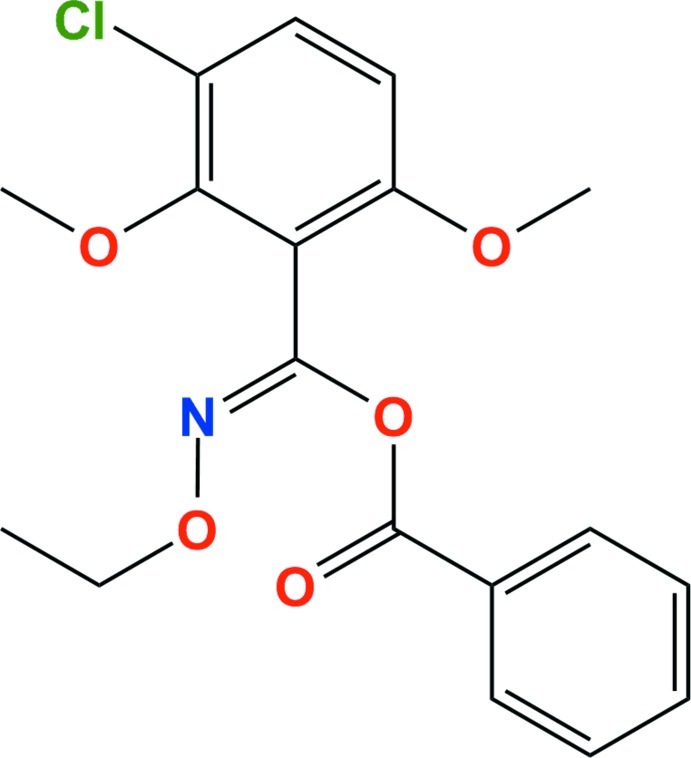


## Experimental

### 

#### Crystal data

C_18_H_18_ClNO_5_
*M* *_r_* = 363.78
Monoclinic, 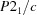

*a* = 9.4262 (10) Å
*b* = 12.9863 (14) Å
*c* = 15.4227 (16) Å
β = 102.843 (2)°
*V* = 1840.7 (3) Å^3^
*Z* = 4
Mo *K*α radiation
μ = 0.23 mm^−1^
*T* = 223 K
0.30 × 0.20 × 0.10 mm

#### Data collection

Bruker APEXII CCD diffractometer
Absorption correction: multi-scan (*SADABS*; Sheldrick, 1996 ▶) *T*_min_ = 0.933, *T*_max_ = 0.977
9736 measured reflections
3606 independent reflections
2298 reflections with *I* > 2σ(*I*)
*R*_int_ = 0.049

#### Refinement

*R*[*F* ^2^ > 2σ(*F* ^2^)] = 0.073
*wR*(*F* ^2^) = 0.138
*S* = 1.10
3606 reflections
229 parameters
H-atom parameters constrained
Δρ_max_ = 0.22 e Å^−3^
Δρ_min_ = −0.30 e Å^−3^

### 

Data collection: *APEX2* (Bruker, 2006 ▶); cell refinement: *SAINT* (Bruker, 2006 ▶); data reduction: *SAINT*; program(s) used to solve structure: *SHELXTL* (Sheldrick, 2008 ▶); program(s) used to refine structure: *SHELXTL*; molecular graphics: *SHELXTL* and *DIAMOND* (Brandenburg, 1998 ▶); software used to prepare material for publication: *SHELXTL*.

## Supplementary Material

Crystal structure: contains datablock(s) I, global. DOI: 10.1107/S1600536812044248/sj5275sup1.cif

Structure factors: contains datablock(s) I. DOI: 10.1107/S1600536812044248/sj5275Isup2.hkl

Supplementary material file. DOI: 10.1107/S1600536812044248/sj5275Isup3.cml

Additional supplementary materials:  crystallographic information; 3D view; checkCIF report

## Figures and Tables

**Table 1:** Hydrogen-bond geometry (Å, °) *Cg*1 and *Cg*2 are the centroids of the C1–C6 and the C13–C18 rings, respectively.

*D*—H⋯*A*	*D*—H	H⋯*A*	*D*⋯*A*	*D*—H⋯*A*
C3—H3⋯O5^i^	0.94	2.57	3.409 (4)	148
C17—H17⋯O1^ii^	0.94	2.63	3.496 (4)	154
C18—H18⋯Cl1^ii^	0.94	2.89	3.716 (3)	147
C7—H7*A*⋯*Cg*1^iii^	0.97	2.90	3.570 (4)	127
C10—H10*A*⋯*Cg*2^iv^	0.98	3.01	3.740 (5)	132

Symmetry codes: (i) 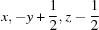; (ii) 
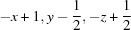
; (iii) 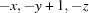; (iv) 
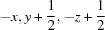
.

**Fig. 1.**
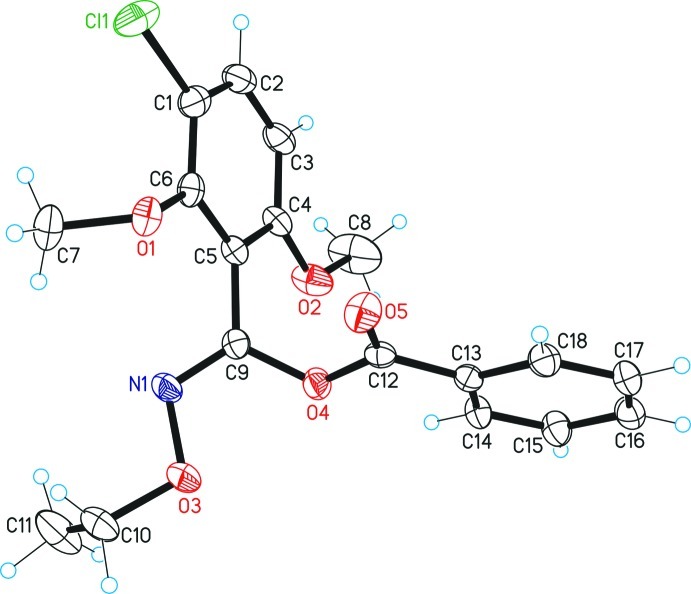
The molecular structure of the title compound, showing displacement ellipsoids drawn at the 30% probability level.

**Fig. 2.**
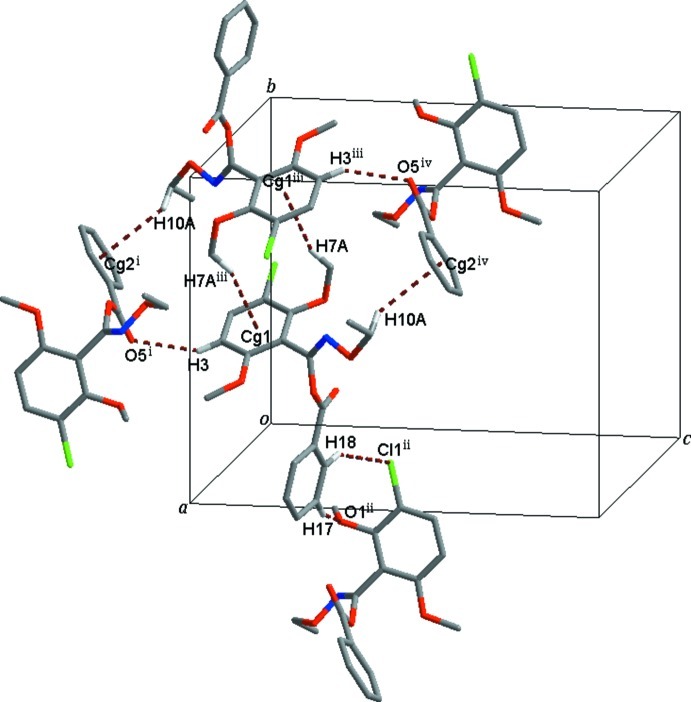
Crystal packing of the title compound with intermolecular C–H···O and C–H···Cl hydrogen bonds and C–H···π interactions shown as dashed lines. H atoms not involved in intermolecular interactions have been omitted for clarity. Cg1 and Cg2 are the centroids of the C1–C6 and the C13–C18 rings, respectively. (Symmetry codes: (i) x, -y + 1/2, z - 1/2; (ii) -x + 1, y - 1/2, -z + 1/2; (iii) -x, -y + 1, -z; (iv) -x, y + 1/2, -z + 1/2.)

## supplementary crystallographic information

### Comment

Benzoximate (IUPAC name: (3-chloro-2,6-dimethoxyphenyl)(ethoxyimino)methyl benzoate) is an acaricide widely used in agriculture targeting ticks and mites (Kim *et al.*, 2007). However, until now its crystal structure has not been reported.

In the title compound (Fig. 1), the two aromatic rings are almost perpendicular to each other with the dihedral angle between them being 85.72 (9)°. Of the two methoxy methyl groups, one (C7) is almost perpendicular to the benzene ring plane [torsion angle C1–C6–O1–C7 = 84.0 (4) °] whereas the other (C8) is nearly parallel to it [torsion angle C3–C4–O2–C8 = 3.8 (5) °]. All the bond distances and bond angles within the molecule agree with values reported in the Cambridge Structural Database (Allen, 2002).

In the crystal, C17—H17···O1 and C18—H18···Cl1 hydrogen bonds generate R^2^_2_(8) rings (Bernstein *et al.,* 1995). Additional C3—H3···O5 hydrogen bonds and C–H···π contacts, Table 1, further link the molecules into a three dimensional network.

### Experimental

The title compound was purchased from the Dr. Ehrenstorfer GmbH Company. X-ray quality single crystals were obtained by slow evaporation of a solution of the title compound in dichloromethane at room temperature.

### Refinement

All H-atoms were positioned geometrically and refined using a riding model with d(C–H) = 0.94 Å, *U*_iso_ = 1.2*U*_eq_(C) for aromatic. d(C–H) = 0.98 Å, *U*_iso_ = 1.2*U*_eq_(C) for methylene, and d(C–H) = 0.97 Å, *U*_iso_ = 1.5*U*_eq_(C) for methyl protons.

### Crystal data

**Table:**

C_18_H_18_ClNO_5_	*F*(000) = 760
*M**_r_* = 363.78	*D*_x_ = 1.313 Mg m^−^^3^
Monoclinic, *P*2_1_/*c*	Mo *K*α radiation, λ = 0.71073 Å
Hall symbol: -P 2ybc	Cell parameters from 2737 reflections
*a* = 9.4262 (10) Å	θ = 2.2–25.6°
*b* = 12.9863 (14) Å	µ = 0.23 mm^−^^1^
*c* = 15.4227 (16) Å	*T* = 223 K
β = 102.843 (2)°	Block, colourless
*V* = 1840.7 (3) Å^3^	0.30 × 0.20 × 0.10 mm
*Z* = 4	

### Data collection

**Table:**

Bruker APEXII CCD diffractometer	3606 independent reflections
Radiation source: fine-focus sealed tube	2298 reflections with *I* > 2σ(*I*)
Graphite monochromator	*R*_int_ = 0.049
φ and ω scans	θ_max_ = 26.0°, θ_min_ = 2.1°
Absorption correction: multi-scan (*SADABS*; Sheldrick, 1996)	*h* = −11→11
*T*_min_ = 0.933, *T*_max_ = 0.977	*k* = −8→16
9736 measured reflections	*l* = −18→19

### Refinement

**Table:**

Refinement on *F*^2^	Primary atom site location: structure-invariant direct methods
Least-squares matrix: full	Secondary atom site location: difference Fourier map
*R*[*F*^2^ > 2σ(*F*^2^)] = 0.073	Hydrogen site location: inferred from neighbouring sites
*wR*(*F*^2^) = 0.138	H-atom parameters constrained
*S* = 1.10	*w* = 1/[σ^2^(*F*_o_^2^) + (0.0351*P*)^2^ + 1.3636*P*] where *P* = (*F*_o_^2^ + 2*F*_c_^2^)/3
3606 reflections	(Δ/σ)_max_ < 0.001
229 parameters	Δρ_max_ = 0.22 e Å^−^^3^
0 restraints	Δρ_min_ = −0.30 e Å^−^^3^

### Special details

**Table:**

Geometry. All e.s.d.'s (except the e.s.d. in the dihedral angle between two l.s. planes) are estimated using the full covariance matrix. The cell e.s.d.'s are taken into account individually in the estimation of e.s.d.'s in distances, angles and torsion angles; correlations between e.s.d.'s in cell parameters are only used when they are defined by crystal symmetry. An approximate (isotropic) treatment of cell e.s.d.'s is used for estimating e.s.d.'s involving l.s. planes.
Refinement. Refinement of *F*^2^ against ALL reflections. The weighted *R*-factor *wR* and goodness of fit *S* are based on *F*^2^, conventional *R*-factors *R* are based on *F*, with *F* set to zero for negative *F*^2^. The threshold expression of *F*^2^ > σ(*F*^2^) is used only for calculating *R*-factors(gt) *etc*. and is not relevant to the choice of reflections for refinement. *R*-factors based on *F*^2^ are statistically about twice as large as those based on *F*, and *R*- factors based on ALL data will be even larger.

### Fractional atomic coordinates and isotropic or equivalent isotropic displacement parameters (Å^2^)

**Table:**

	*x*	*y*	*z*	*U*_iso_*/*U*_eq_	
Cl1	0.31473 (12)	0.56710 (7)	0.07179 (7)	0.0649 (3)	
O1	0.1499 (2)	0.42350 (16)	0.16181 (13)	0.0405 (6)	
O2	0.1097 (3)	0.15458 (19)	−0.04964 (16)	0.0583 (7)	
O3	−0.1289 (3)	0.17796 (19)	0.17061 (16)	0.0578 (7)	
O4	0.0986 (2)	0.12350 (16)	0.12436 (14)	0.0404 (6)	
O5	0.2986 (3)	0.18389 (17)	0.21829 (15)	0.0484 (6)	
N1	−0.0722 (3)	0.2521 (2)	0.12132 (17)	0.0415 (7)	
C1	0.2563 (4)	0.4441 (3)	0.0356 (2)	0.0431 (9)	
C2	0.2902 (4)	0.4053 (3)	−0.0402 (2)	0.0488 (9)	
H2	0.3459	0.4449	−0.0714	0.059*	
C3	0.2430 (4)	0.3084 (3)	−0.0708 (2)	0.0477 (9)	
H3	0.2657	0.2823	−0.1229	0.057*	
C4	0.1622 (3)	0.2502 (3)	−0.0243 (2)	0.0396 (8)	
C5	0.1277 (3)	0.2883 (2)	0.0537 (2)	0.0328 (7)	
C6	0.1751 (3)	0.3869 (2)	0.0833 (2)	0.0355 (8)	
C7	0.0273 (4)	0.4934 (3)	0.1513 (2)	0.0539 (10)	
H7A	0.0326	0.5436	0.1055	0.081*	
H7B	0.0300	0.5287	0.2070	0.081*	
H7C	−0.0627	0.4548	0.1342	0.081*	
C8	0.1477 (6)	0.1095 (4)	−0.1262 (3)	0.0996 (19)	
H8A	0.1149	0.1539	−0.1773	0.149*	
H8B	0.1014	0.0427	−0.1378	0.149*	
H8C	0.2525	0.1014	−0.1153	0.149*	
C9	0.0461 (3)	0.2231 (2)	0.10433 (19)	0.0331 (7)	
C10	−0.2591 (5)	0.2193 (3)	0.1924 (3)	0.0682 (12)	
H10A	−0.2394	0.2897	0.2147	0.082*	
H10B	−0.2832	0.1780	0.2404	0.082*	
C11	−0.3862 (5)	0.2206 (4)	0.1162 (3)	0.0910 (16)	
H11A	−0.3699	0.2706	0.0727	0.137*	
H11B	−0.4727	0.2393	0.1368	0.137*	
H11C	−0.3991	0.1529	0.0891	0.137*	
C12	0.2377 (3)	0.1144 (2)	0.17402 (19)	0.0328 (7)	
C13	0.2986 (3)	0.0111 (2)	0.16451 (19)	0.0311 (7)	
C14	0.2277 (3)	−0.0602 (2)	0.1025 (2)	0.0390 (8)	
H14	0.1367	−0.0443	0.0658	0.047*	
C15	0.2915 (4)	−0.1537 (3)	0.0952 (2)	0.0466 (9)	
H15	0.2432	−0.2023	0.0539	0.056*	
C16	0.4257 (4)	−0.1772 (3)	0.1479 (2)	0.0479 (9)	
H16	0.4684	−0.2415	0.1422	0.058*	
C17	0.4976 (4)	−0.1070 (3)	0.2091 (2)	0.0487 (9)	
H17	0.5895	−0.1230	0.2447	0.058*	
C18	0.4341 (4)	−0.0130 (3)	0.2177 (2)	0.0412 (8)	
H18	0.4824	0.0350	0.2597	0.049*	

### Atomic displacement parameters (Å^2^)

**Table:**

	*U*^11^	*U*^22^	*U*^33^	*U*^12^	*U*^13^	*U*^23^
Cl1	0.0716 (7)	0.0457 (5)	0.0686 (7)	−0.0158 (5)	−0.0033 (5)	0.0124 (5)
O1	0.0476 (14)	0.0375 (12)	0.0331 (12)	0.0087 (11)	0.0017 (10)	−0.0030 (10)
O2	0.0666 (19)	0.0633 (16)	0.0548 (16)	−0.0204 (14)	0.0342 (13)	−0.0286 (13)
O3	0.0556 (17)	0.0620 (16)	0.0683 (17)	0.0102 (13)	0.0405 (14)	0.0162 (14)
O4	0.0319 (13)	0.0349 (12)	0.0532 (14)	0.0003 (10)	0.0072 (11)	−0.0047 (11)
O5	0.0540 (16)	0.0413 (14)	0.0443 (14)	−0.0034 (12)	−0.0011 (12)	−0.0114 (12)
N1	0.0387 (17)	0.0502 (17)	0.0413 (16)	0.0052 (14)	0.0209 (13)	0.0051 (14)
C1	0.037 (2)	0.042 (2)	0.045 (2)	−0.0017 (16)	−0.0029 (17)	0.0071 (17)
C2	0.035 (2)	0.061 (2)	0.051 (2)	−0.0006 (18)	0.0107 (17)	0.017 (2)
C3	0.041 (2)	0.063 (2)	0.044 (2)	0.0027 (19)	0.0217 (17)	0.0002 (19)
C4	0.0301 (18)	0.049 (2)	0.0417 (19)	−0.0001 (16)	0.0116 (15)	−0.0066 (17)
C5	0.0265 (18)	0.0394 (18)	0.0324 (17)	0.0022 (14)	0.0061 (13)	−0.0020 (15)
C6	0.0343 (19)	0.0370 (18)	0.0309 (17)	0.0077 (15)	−0.0020 (14)	0.0005 (15)
C7	0.064 (3)	0.046 (2)	0.051 (2)	0.0208 (19)	0.0107 (19)	−0.0014 (18)
C8	0.130 (5)	0.099 (4)	0.093 (4)	−0.037 (3)	0.075 (3)	−0.063 (3)
C9	0.0339 (19)	0.0333 (17)	0.0318 (17)	0.0007 (15)	0.0065 (15)	−0.0051 (14)
C10	0.063 (3)	0.079 (3)	0.077 (3)	0.010 (2)	0.047 (2)	0.011 (2)
C11	0.054 (3)	0.140 (5)	0.088 (3)	0.007 (3)	0.036 (3)	0.001 (3)
C12	0.0356 (19)	0.0361 (18)	0.0292 (17)	−0.0038 (16)	0.0124 (15)	0.0001 (15)
C13	0.0314 (18)	0.0348 (17)	0.0282 (16)	−0.0014 (14)	0.0089 (14)	−0.0022 (14)
C14	0.0313 (19)	0.0422 (19)	0.0415 (18)	0.0051 (16)	0.0040 (15)	−0.0053 (16)
C15	0.041 (2)	0.044 (2)	0.053 (2)	0.0018 (17)	0.0070 (18)	−0.0115 (17)
C16	0.041 (2)	0.044 (2)	0.063 (2)	0.0039 (17)	0.0187 (19)	−0.0032 (18)
C17	0.0304 (19)	0.052 (2)	0.061 (2)	0.0066 (17)	0.0061 (17)	0.0089 (19)
C18	0.034 (2)	0.047 (2)	0.0396 (19)	−0.0028 (16)	0.0003 (15)	0.0001 (16)

### Geometric parameters (Å, º)

**Table:**

Cl1—C1	1.740 (3)	C7—H7C	0.9700
O1—C6	1.370 (4)	C8—H8A	0.9700
O1—C7	1.450 (4)	C8—H8B	0.9700
O2—C4	1.361 (4)	C8—H8C	0.9700
O2—C8	1.433 (4)	C10—C11	1.481 (6)
O3—N1	1.404 (3)	C10—H10A	0.9800
O3—C10	1.446 (4)	C10—H10B	0.9800
O4—C12	1.368 (4)	C11—H11A	0.9700
O4—C9	1.395 (4)	C11—H11B	0.9700
O5—C12	1.198 (3)	C11—H11C	0.9700
N1—C9	1.259 (4)	C12—C13	1.479 (4)
C1—C2	1.374 (5)	C13—C14	1.391 (4)
C1—C6	1.389 (5)	C13—C18	1.392 (4)
C2—C3	1.382 (5)	C14—C15	1.371 (4)
C2—H2	0.9400	C14—H14	0.9400
C3—C4	1.382 (5)	C15—C16	1.377 (5)
C3—H3	0.9400	C15—H15	0.9400
C4—C5	1.404 (4)	C16—C17	1.377 (5)
C5—C6	1.398 (4)	C16—H16	0.9400
C5—C9	1.478 (4)	C17—C18	1.379 (5)
C7—H7A	0.9700	C17—H17	0.9400
C7—H7B	0.9700	C18—H18	0.9400
			
C6—O1—C7	114.1 (2)	N1—C9—C5	121.8 (3)
C4—O2—C8	117.9 (3)	O4—C9—C5	116.6 (3)
N1—O3—C10	108.5 (3)	O3—C10—C11	113.4 (3)
C12—O4—C9	116.9 (2)	O3—C10—H10A	108.9
C9—N1—O3	111.7 (3)	C11—C10—H10A	108.9
C2—C1—C6	120.9 (3)	O3—C10—H10B	108.9
C2—C1—Cl1	119.5 (3)	C11—C10—H10B	108.9
C6—C1—Cl1	119.6 (3)	H10A—C10—H10B	107.7
C1—C2—C3	120.5 (3)	C10—C11—H11A	109.5
C1—C2—H2	119.8	C10—C11—H11B	109.5
C3—C2—H2	119.8	H11A—C11—H11B	109.5
C2—C3—C4	119.6 (3)	C10—C11—H11C	109.5
C2—C3—H3	120.2	H11A—C11—H11C	109.5
C4—C3—H3	120.2	H11B—C11—H11C	109.5
O2—C4—C3	123.9 (3)	O5—C12—O4	122.2 (3)
O2—C4—C5	115.4 (3)	O5—C12—C13	126.3 (3)
C3—C4—C5	120.7 (3)	O4—C12—C13	111.5 (3)
C6—C5—C4	119.0 (3)	C14—C13—C18	119.6 (3)
C6—C5—C9	121.2 (3)	C14—C13—C12	122.3 (3)
C4—C5—C9	119.8 (3)	C18—C13—C12	118.1 (3)
O1—C6—C1	120.4 (3)	C15—C14—C13	119.6 (3)
O1—C6—C5	120.0 (3)	C15—C14—H14	120.2
C1—C6—C5	119.4 (3)	C13—C14—H14	120.2
O1—C7—H7A	109.5	C14—C15—C16	120.6 (3)
O1—C7—H7B	109.5	C14—C15—H15	119.7
H7A—C7—H7B	109.5	C16—C15—H15	119.7
O1—C7—H7C	109.5	C15—C16—C17	120.4 (3)
H7A—C7—H7C	109.5	C15—C16—H16	119.8
H7B—C7—H7C	109.5	C17—C16—H16	119.8
O2—C8—H8A	109.5	C16—C17—C18	119.6 (3)
O2—C8—H8B	109.5	C16—C17—H17	120.2
H8A—C8—H8B	109.5	C18—C17—H17	120.2
O2—C8—H8C	109.5	C17—C18—C13	120.2 (3)
H8A—C8—H8C	109.5	C17—C18—H18	119.9
H8B—C8—H8C	109.5	C13—C18—H18	119.9
N1—C9—O4	121.2 (3)		
			
C10—O3—N1—C9	176.3 (3)	O3—N1—C9—C5	−179.5 (3)
C6—C1—C2—C3	0.5 (5)	C12—O4—C9—N1	−126.9 (3)
Cl1—C1—C2—C3	−179.0 (3)	C12—O4—C9—C5	59.6 (3)
C1—C2—C3—C4	−0.5 (5)	C6—C5—C9—N1	58.3 (4)
C8—O2—C4—C3	3.8 (5)	C4—C5—C9—N1	−123.5 (3)
C8—O2—C4—C5	−176.9 (4)	C6—C5—C9—O4	−128.2 (3)
C2—C3—C4—O2	179.3 (3)	C4—C5—C9—O4	50.0 (4)
C2—C3—C4—C5	0.0 (5)	N1—O3—C10—C11	74.9 (4)
O2—C4—C5—C6	−178.7 (3)	C9—O4—C12—O5	19.6 (4)
C3—C4—C5—C6	0.6 (5)	C9—O4—C12—C13	−160.0 (2)
O2—C4—C5—C9	3.0 (4)	O5—C12—C13—C14	−171.1 (3)
C3—C4—C5—C9	−177.6 (3)	O4—C12—C13—C14	8.4 (4)
C7—O1—C6—C1	84.0 (4)	O5—C12—C13—C18	6.6 (5)
C7—O1—C6—C5	−100.4 (3)	O4—C12—C13—C18	−173.9 (3)
C2—C1—C6—O1	175.7 (3)	C18—C13—C14—C15	0.7 (5)
Cl1—C1—C6—O1	−4.7 (4)	C12—C13—C14—C15	178.3 (3)
C2—C1—C6—C5	0.1 (5)	C13—C14—C15—C16	−0.8 (5)
Cl1—C1—C6—C5	179.7 (2)	C14—C15—C16—C17	0.2 (5)
C4—C5—C6—O1	−176.3 (3)	C15—C16—C17—C18	0.4 (5)
C9—C5—C6—O1	1.9 (4)	C16—C17—C18—C13	−0.5 (5)
C4—C5—C6—C1	−0.7 (4)	C14—C13—C18—C17	0.0 (5)
C9—C5—C6—C1	177.5 (3)	C12—C13—C18—C17	−177.7 (3)
O3—N1—C9—O4	7.3 (4)		

### Hydrogen-bond geometry (Å, º)

*Cg*1 and *Cg*2 are the centroids of the C1–C6 and the C13–C18 rings, respectively.

**Table:**

*D*—H···*A*	*D*—H	H···*A*	*D*···*A*	*D*—H···*A*
C3—H3···O5^i^	0.94	2.57	3.409 (4)	148
C17—H17···O1^ii^	0.94	2.63	3.496 (4)	154
C18—H18···Cl1^ii^	0.94	2.89	3.716 (3)	147
C7—H7*A*···*Cg*1^iii^	0.97	2.90	3.570 (4)	127
C10—H10*A*···*Cg*2^iv^	0.98	3.01	3.740 (5)	132

Symmetry codes: (i) *x*, −*y*+1/2, *z*−1/2; (ii) −*x*+1, *y*−1/2, −*z*+1/2; (iii) −*x*, −*y*+1, −*z*; (iv) −*x*, *y*+1/2, −*z*+1/2.

## References

[bb1] Allen, F. H. (2002). *Acta Cryst.* B**58**, 380–388. 10.1107/s0108768102003890 12037359

[bb2] Bernstein, J., Davis, R. E., Shimoni, L. & Chang, N.-L. (1995). *Angew. Chem. Int. Ed. Engl.* **34**, 1555–1573.

[bb3] Brandenburg, K. (1998). *DIAMOND* Crystal Impact GbR, Bonn, Germany.

[bb4] Bruker (2006). *APEX2* and *SAINT* Bruker AXS Inc., Madison, Wisconsin, USA.

[bb5] Kim, Y.-J., Lee, S.-W., Cho, J.-R., Park, H.-M. & Ahn, Y.-J. (2007). *J. Asia Pac. Entomol.* **10**, 165–170.

[bb6] Sheldrick, G. M. (1996). *SADABS* University of Göttingen, Germany.

[bb7] Sheldrick, G. M. (2008). *Acta Cryst.* A**64**, 112–122. 10.1107/S0108767307043930 18156677

